# Relationship between drug application and mortality rate in Chinese older coronary artery disease/chronic heart failure patients with and without low glomerular filtration rate

**DOI:** 10.1186/s40360-019-0320-z

**Published:** 2019-07-26

**Authors:** Shihui Fu, Ping Ping, Ping Ye, Leiming Luo

**Affiliations:** 1 Department of Geriatric Cardiology, Chinese People’s Liberation Army General Hospital, Beijing, China; 2 Department of Cardiology and Hainan Branch, Chinese People’s Liberation Army General Hospital, Beijing, China; 3 Department of Pharmacentical Care, Chinese People’s Liberation Army General Hospital, Beijing, China

**Keywords:** Chronic heart failure, Coronary artery disease, Drug application, Older patients, Mortality rate

## Abstract

**Background:**

This analysis was designed to investigate the relationship between drug application and mortality rate in Chinese older coronary artery disease (CAD)/chronic heart failure (CHF) patients with and without low glomerular filtration rate (GFR).

**Methods:**

All 1050 Chinese hospitalized patients with diagnosed CAD were included in this analysis, and Cox Regression was used to analyze the relationship between drug application and mortality rate after multivariate adjustment. Low GFR was defined as GFR < 60 ml/min/1.73m^2^.

**Results:**

There were 372 patients (35.4%) with low GFR in patients with CAD (1050 patients), and 168 patients (51.4%) in patients with CHF (327 patients). In CAD patients without low GFR, clopidogrel [*P* = 0.028, odds ratio (OR): 0.620, 95% confidence interval (CI): 0.404–0.951] rather than aspirin (*P* = 0.173) was significantly associated with lower mortality rate. Statins (*P* < 0.001, OR: 0.287, 95% CI: 0.180–0.456) were significantly associated with lower mortality rate. In CAD patients with low GFR, aspirin, clopidogrel and statins had no significant relationship with mortality rate (*P* > 0.05 for all). In CHF patients without low GFR, statins were significantly associated with lower mortality rate (*P* < 0.001, OR: 0.220, 95% CI: 0.098–0.490). In CHF patients with low GFR, statins had no significant relationship with mortality rate (*P* > 0.05 for all).

**Conclusion:**

Clopidogrel but not aspirin was beneficial in Chinese older CAD patients without low GFR rather than those with low GFR, and statins benefited for Chinese older CAD/CHF patients without low GFR rather than those with low GFR. These discoveries might offer some help for the therapy of Chinese older patients with cardiovascular/renal diseases.

## Background

Renal function decline is very common and predicts adverse outcome in older patients with coronary artery disease (CAD) and chronic heart failure (CHF). Moreover, renal function decline significantly accelerates disease progression, intensively increase mortality rate and severely complicate prognostic effects in older patients with CAD/CHF [1]. Drugs, such as anti-platelet drugs, beta-blockers, calcium channel blockers (CCBs), nitrates, angiotensin-converting enzyme inhibitors/angiotensin receptor blockers (ACEI/ARBs), statins and digoxin, have gained widespread acceptance as the principal therapies for CAD/CHF [2, 3]. However, prognostic effects of these drugs might be quite different between older CAD/CHF patients with and without renal function decline [1].

There have been several studies about the relationship between these drugs and mortality rate [2, 3]. However, as these studies often excluded Chinese older patients with renal function decline, there has been no full evidence about the relationship between these drugs and mortality rate. As a retrospective review of medical records, the goal of this analysis was to investigate the relationship between drug application and mortality rate in Chinese older CAD/CHF patients with and without low glomerular filtration rate (GFR).

## Methods

### Study population

There were 1050 Chinese hospitalized older patients with diagnosed CAD in Chinese People’s Liberation Army General Hospital enrolled in this study. These patients had a median age of 86 (60–104) years, and 80.8% (848 patients) were over 80 years old. Based on medical histories, typical angina, cardiac markers and specific tests, such as electrocardiogram (resting/exercise), echocardiography, radionuclide imaging, computed tomography angiography and coronary angiography, CAD was diagnosed by chief physicians according to American College of Cardiology (ACC)/American Heart Association (AHA)/European Society of Cardiology (ESC) guidelines [4–6]. Exclusion criteria: 1) severe aortic stenosis; 2) anticipated cardiac transplantation; and 3) left ventricular assist device. The study was approved by Ethics Committee of Chinese People’s Liberation Army General Hospital (Beijing, China; Number: 038). Written consent was obtained from patients when admission and Helsinki declaration was followed by the study.

### Baseline characteristics

Baseline characteristics available for this analysis included demographics (age and gender), physical examination [height, weight, heart rate, systolic and diastolic blood pressure (SBP and DBP)], laboratory measurements [hemoglobin, albumin, high-density lipoprotein cholesterol (HDL-C), low-density lipoprotein cholesterol (LDL-C), triglyceride and fasting plasma glucose (FPG)]. GFR was calculated by a modifying Modification of Diet in Renal Disease (MDRD) equation based on the data from Chinese patients: 175 × serum creatinine (mg/dl) ^-1.234^ × age (year)^-0.179^ × 0.79 (if female) [7, 8]. Low GFR was defined as GFR < 60 ml/min/1.73m^2^. Atrial fibrillation (AF) and CHF were diagnosed by chief physicians on the ground of ACC/AHA/ESC guidelines [9, 10]. Patients with SBP ≥ 140 mmHg, DBP ≥ 90 mmHg or receiving anti-hypertensive drugs were defined as having hypertension.

### Data management

Data were recorded in the database by trained doctors with full experience, and logistic check was performed by other doctors to ensure the accuracy of database. Aspirin, clopidogrel, beta-blockers, such as metoprolol, atenolol and bisoprolol, CCBs, such as nifedipine, amlodipine and felodipine, nitrates, such as and isosorbide mononitrate, ACEI/ARBs, such as captopril, enalapril, benazepril, fosinopril, losartan, valsartan and irbesartan, statins, such as simvastatin, pravastatin, atorvastatin and rosuvastatin, and digoxin were recorded in the database.

### Mortality rate

Given the obviously high incidence of multiple organ failure in older patients as well as the priority of all-cause mortality in outcome studies, all-cause mortality was the predefined end point for this study. This analysis had a mean follow-up period of 417 days [median 319 (185–557) days]. No patient was lost to follow-up. Death was ascertained from death record, a legal document including time, site and other information.

### Statistical analyses

Continuous variables were described by mean and standard deviation (normal distribution) or median and interquartile range (skewed distribution). Continuous variables were compared by Student’s t test (normal distribution) or Mann–Whitney U test (skewed distribution). Categorical variables were described by number and percentage, and compared by chi-square test. Relationship of drugs with survival time was bivariately evaluated with Kaplan-Meier analysis (log-rank). Relationship of drugs with mortality rate were multivariately adjusted by age, gender, body mass index (BMI), CAD, CHF, AF, hypertension, heart rate, SBP, DBP, hemoglobin, albumin, GFR, FPG, triglyceride, LDL-C and HDL-C using Cox Regression (Enter). Enter is the most commonly used in different methods of Cox Regression, such as Enter, Forward conditional, Forward LR, Forward Wald, Backward conditional, Backward LR and Backward Wald. Statistical analyses were performed with Statistic Package for Social Science (SPSS) version 17 (SPSS Inc., Chicago, USA).

## Results

### Utilization ratios of drugs in CAD/CHF patients

There were 372 patients (35.4%) with low GFR in patients with CAD (1050 patients), and 168 patients (51.4%) in patients with CHF (327 patients). Basic features of patients with and without low GFR were described in Table 1. As shown in Table 2, aspirin, clopidogrel, beta-blockers, CCBs, nitrates, ACEI/ARBs, statins and digoxin gained high utilization ratios regardless of these patients with low GFR or not. Except CCBs (*P* < 0.05), there was no difference in utilization ratios of other drugs between these patients with and without low GFR (*P* > 0.05 for all).

**Table 1:** Basic features of older CAD patients with and without low GFR

Features	All	Without low GFR	With low GFR	*P* value
Age (year) ^a^	86.0 (81.0–89.0)	85.0 (80.0–89.0)	87.0 (83.0–90.0)	< 0.001
Males (%)	937 (89.2)	617 (91.0)	320 (86.0)	0.013
BMI (kg/m^2^) mean (SD)	23.9 (3.5)	23.9 (3.5)	23.9 (3.5)	0.601
CHF (%)	327 (31.1)	159 (23.5)	168 (45.2)	< 0.001
AF (%)	219 (20.9)	131 (19.3)	88 (23.7)	0.098
Hypertension (%)	839 (79.9)	516 (76.1)	323 (86.8)	< 0.001
Heart rate (bpm) ^a^	72.0 (64.0–80.0)	72.0 (64.8–80.0)	72.0 (64.0–80.0)	0.723
SBP (mmHg) ^a^	132.4 (123.8–142.0)	131.8 (123.0–141.6)	133.4 (125.4–143.0)	0.053
DBP (mmHg) ^a^	69.0 (63.7–74.3)	69.2 (64.3–74.3)	68.8 (63.0–74.3)	0.294
Hemoglobin (g/L) ^a^	124.0 (109.0–137.0)	128.0 (114.8–140.0)	113.0 (101.0–128.0)	< 0.001
Albumin (g/L) ^a^	38.3 (35.3–40.8)	38.8 (35.9–41.2)	37.7 (34.6–39.9)	< 0.001
GFR (ml/min/1.73m^2^) ^a^	69.2 (53.3–82.3)	78.6 (70.4–89.0)	47.2 (35.0–54.4)	< 0.001
FPG (mmol/L) ^a^	5.4 (4.8–6.2)	5.3 (4.8–6.1)	5.4 (4.8–6.3)	0.357
Triglyceride (mmol/L) ^a^	1.2 (0.9–1.8)	1.2 (0.9–1.6)	1.4 (1.0–2.0)	< 0.001
LDL-C (mmol/L) ^a^	2.1 (1.7–2.6)	2.2 (1.8–2.7)	2.0 (1.6–2.5)	0.001
HDL-C (mmol/L) ^a^	1.1 (0.9–1.3)	1.1 (0.9–1.3)	1.0 (0.8–1.2)	< 0.001

^a^Median (interquartile range)

*Abbreviations*: *CAD* Coronary artery disease, *BMI* Body mass index, *CHF* Chronic heart failure, *AF* Atrial fibrillation, *SBP* Systolic blood pressure, *DBP* Diastolic blood pressure, *GFR* Glomerular filtration rate, *FPG* Fasting plasma glucose, *LDL-C* Low density lipoprotein cholesterol, *HDL-C* High density lipoprotein cholesterol, *SD* Standard deviation

**Table 2:** Utilization ratios of drugs in older CAD/CHF patients with and without low GFR

Diseases	Drugs	All	Without low GFR	With low GFR	*P* value^*^
CAD	Aspirin (%)	537 (51.1)	355 (52.4)	182 (48.9)	0.287
	Clopidogrel (%)	622 (59.2)	409 (60.3)	213 (57.3)	0.333
	Beta-blockers (%)	705 (67.1)	445 (65.6)	260 (69.9)	0.160
	CCBs (%)	664 (63.2)	410 (60.5)	254 (68.3)	0.012
	Nitrates (%)	871 (83.0)	558 (82.3)	313 (84.1)	0.449
	ACEI/ARBs (%)	530 (50.5)	346 (51.0)	184 (49.5)	0.626
	Statins (%)	693 (66.0)	453 (66.8)	240 (64.5)	0.452
CHF	Beta-blockers (%)	252 (77.1)	117 (73.6)	135 (80.4)	0.145
	ACEI/ARBs (%)	178 (54.4)	92 (57.9)	86 (51.2)	0.626
	Statins (%)	214 (65.4)	105 (66.0)	109 (64.9)	0.826
	Digoxin (%)	168 (51.4)	76 (47.8)	92 (54.8)	0.208

*Comparing utilization ratios of drugs between patients with and without low GFR

*Abbreviations*: *ACEI/ARBs* Renin-angiotensin-aldosterone system inhibitors/angiotensin receptor blockers, *CAD* Coronary artery disease, *CHF* Chronic heart failure, *CCBs* Calcium channel blockers, *GFR* Glomerular filtration rate

### Prognostic effects of drugs in CAD patients

In CAD patients without low GFR (Table 3), patients with aspirin (*P* = 0.023) or clopidogrel (*P* = 0.008) had significantly longer survival time than those without them. However, clopidogrel [*P* = 0.028, odds ratio (OR): 0.620, 95% confidence interval (CI): 0.404–0.951] rather than aspirin (*P* = 0.173) was significantly associated with lower mortality rate after adjustment shown above. Patients with ACEI/ARBs (*P* = 0.033) or statins (*P* < 0.001) had significantly longer survival time than those without them. However, statins (*P* < 0.001, OR: 0.287, 95% CI: 0.180–0.456) rather than ACEI/ARBs (*P* = 0.426) were significantly associated with lower mortality rate after adjustment. Beta-blockers, CCBs and nitrates had no significant relationship with survival time and mortality rate (*P* > 0.05 for all).

**Table 3:** Prognostic effects of drugs in CAD patients with and without low GFR

Drugs	Without low GFR				With low GFR			
	Survival time (days)		*P* value^*^	*P* value^**^	Survival time (days)		*P* value^*^	*P* value^**^
	No use	Use			No use	Use		
Aspirin	883 (828–937)	1086 (1019–1152)	0.023	0.173	900 (785–1015)	813 (718–908)	0.349	0.445
Clopidogrel	946 (877–1015)	1088 (1024–1153)	0.008	0.028	942 (835–1050)	739 (646–832)	0.718	0.871
Beta-blockers	964 (915–1014)	1024 (961–1088)	0.155	0.436	1118 (1017–1220)	724 (635–813)	0.013	0.388
CCBs	899 (840–958)	1062 (996–1129)	0.209	0.499	711 (602–821)	910 (808–1013)	0.432	0.223
Nitrates	903 (841–965)	1038 (981–1095)	0.246	0.692	908 (760–1056)	867 (769–966)	0.225	0.460
ACEI/ARBs	1026 (947–1105)	1077 (1007–1148)	0.033	0.426	816 (698–934)	835 (745–926)	0.046	0.671
Statins	903 (821–986)	1136 (1077–1194)	< 0.001	< 0.001	796 (656–937)	811 (729–892)	0.002	0.225

^*^Relationship of drugs with survival time was bivariately evaluated with Kaplan-Meier analysis; ^**^relationship of drugs with mortality rate was multivariately adjusted by age, gender, body mass index, CAD, CHF, atrial fibrillation, hypertension, heart rate, systolic blood pressure, diastolic blood pressure, hemoglobin, albumin, glomerular filtration rate, fasting plasma glucose, triglyceride, low density lipoprotein cholesterol and high density lipoprotein cholesterol using Cox Regression

*Abbreviations*: *ACEI/ARBs* Renin-angiotensin-aldosterone system inhibitors/angiotensin receptor blockers, *CAD* Coronary artery disease, *CHF* Chronic heart failure, *CCBs* Calcium channel blockers, *GFR* Glomerular filtration rate

In CAD patients with low GFR (Table 3), patients with beta-blockers (*p* = 0.013), ACEI/ARBs (*p* = 0.046) or statins (*P* = 0.002) had significantly longer survival time than those without them. However, beta-blockers (*p* = 0.388), ACEI/ARBs (*p* = 0.671) and statins (*P* = 0.225) were not significantly associated with lower mortality rate after adjustment. Aspirin, clopidogrel, CCBs and nitrates had no significant relationship with survival time and mortality rate (*P* > 0.05 for all).

### Prognostic effects of drugs in CHF patients

In CHF patients without low GFR (Table 4), statins were significantly associated with longer survival time (*P* < 0.001) and lower mortality rate (*P* < 0.001, OR: 0.220, 95% CI: 0.098–0.490). Patients with digoxin had significantly shorter survival time than those without them (*P* = 0.004). However, digoxin was not significantly associated with higher mortality rate after adjustment (*P* = 0.519). Beta-blockers and ACEI/ARBs had no significant relationship with survival time and mortality rate (*P* > 0.05 for all).

**Table 4:** Prognostic effects of drugs in CHF patients with and without low GFR

Drugs	Without low GFR				With low GFR			
	Survival time (days)		*P* value	*P* value	Survival time (days)		*P* value	*P* value
	No use	Use			No use	Use		
Beta-blockers	806 (652–959)	774 (686–863)	0.633	0.866	928 (705–1151)	603 (492–714)	0.742	0.885
ACEI/ARBs	791 (670–912)	805 (697–913)	0.370	0.911	634 (461–808)	702 (556–847)	0.125	0.426
Statins	581 (437–724)	889 (807–971)	< 0.001	< 0.001	681 (457–904)	639 (511–767)	0.203	0.641
Digoxin	890 (801–998)	691 (567–816)	0.004	0.519	652 (531–774)	626 (457–795)	0.037	0.095

^*^Relationship of drugs with survival time was bivariately evaluated with Kaplan-Meier analysis; ^**^relationship of drugs with mortality rate was multivariately adjusted by age, gender, body mass index, CAD, CHF, atrial fibrillation, hypertension, heart rate, systolic blood pressure, diastolic blood pressure, hemoglobin, albumin, glomerular filtration rate, fasting plasma glucose, triglyceride, low density lipoprotein cholesterol and high density lipoprotein cholesterol using Cox Regression

*Abbreviations*: *ACEI/ARBs* Renin-angiotensin-aldosterone system inhibitors/angiotensin receptor blockers, *CAD* Coronary artery disease, *CHF* Chronic heart failure, *GFR* Glomerular filtration rate

In CHF patients with low GFR (Table 4), patients with digoxin (*P* = 0.037) had significantly shorter survival time than those without them. However, digoxin (*P* = 0.095) was not significantly associated with higher mortality rate after adjustment. Beta-blockers, ACEI/ARBs and statins had no significant relationship with survival time and mortality rate (*P* > 0.05 for all).

## Discussion

In the recent years, cardiorenal syndrome (CRS) has been paid close attention all over the world [1]. On the one hand, cardiovascular diseases contribute to the development of renal diseases; on the other hand, renal diseases accelerate the progression of cardiovascular diseases. Drugs, such as anti-platelet drugs, beta-blockers, CCBs, nitrates, ACEI/ARBs, statins and digoxin, were commonly used in the therapy for CAD or CHF [2, 3]. This analysis not only investigated their utilization ratio, but also evaluated their relationship with survival time and mortality rate in Chinese older CAD/CHF patients with and without low GFR.

Anti-platelet drugs have been recommended as fundamental treatment for CAD by ACC/AHA guidelines [4, 5]. Bleeding risk is obviously elevated in CAD patients with renal function decline, and the balance between benefit and harm related to anti-platelet therapies remains undefined. It has been confirmed in previous studies that patients with aspirin had no decreased GFR or major bleeding [11]. The Hypertension Optimal Treatment (HOT) study has shown that aspirin more significantly reduced major cardiovascular events and mortality rate in patients with renal function decline than those with normal renal function [12]. This analysis observed that clopidogrel but not aspirin had significant relationship with reduced mortality rate in Chinese older CAD patients without low GFR rather than those with low GFR. This might be partly explained by uremic-induced platelet dysfunction inherently seen in patients with renal function decline.

Beta-blockers have been found to improve clinical symptoms in randomized controlled trials (RCTs) and considered as chronic therapy for CAD by ACC/AHA guidelines [4, 5]. Meanwhile, beta-blockers have been suggested to reduce the risk of HF death in RCTs and recommended to treat stable and symptomatic HF by ACC/AHA/ESC guidelines [3]. However, due to adverse effects including possible hypotension and conflicting outcome, beta-blockers have been underused by clinical doctors in patients with renal function decline [13]. This analysis suggested that beta-blockers had no significant relationship with mortality rate in Chinese older CAD/CHF patients with and without low GFR. Neutral effects were similarly observed for CCBs in this analysis. CCBs and nitrates have been realized to improve clinical symptoms in RCTs and recommend to treat CAD intolerable to beta-blockers by ACC/AHA guidelines [4, 5]. Hypotension was a main limiting factor of applying nitrates in CAD or CHF patients with renal function decline [11]. Nitrates have the potential to reduce death risk in RCTs and recommended as a supplement to treat symptomatic HF with or intolerable to ACEI/ARBs by ACC/AHA/ESC guidelines [3]. No matter whether these patients had low GFR or not, this analysis found that nitrate application had no significant relationship with mortality rate in Chinese older CAD/CHF patients.

ACEI/ARBs have been demonstrated to reduce mortality rate in RCTs and recommended to treat CAD and HF by ACC/AHA/ESC guidelines [3–6]. Even though with this evidence-based consensus of cardiovascular/renal protection with ACEI/ARBs, there are still many experts worrying that hypotension, hyperkalemia and renal function decline are ominous signs of poor outcome, especially in older patients with low GFR [14]. In spite of advanced age and decreased GFR in patients belonged to this analysis, there was significant relationship between ACEI/ARB application and survival time in CAD patients without regard to the existence of low GFR or not. However, ACEI/ARB application had no significant relationship with mortality rate in older patients with CAD/CHF after adjustment in this analysis.

Statins have the potential to play significant role in cardiovascular protection in both patients with and without renal function decline. Statins have been recommended as conventional treatment for CAD by ACC/AHA guidelines [4, 5]. However, it was controversial if statins could have positive effects on mortality rate in CAD/CHF patients with renal function decline [15]. In patients with low GFR rather than those without low GFR, statin application had significant relationship with reduced mortality rate in Chinese older patients with CAD/CHF after adjustment in this analysis.

The risk of toxicity from their narrow therapeutic window, as well as related adverse outcome, might be increased in patients with renal function decline due to the excretion of digoxin from the kidney [13]. Previous study has shown that there was increased mortality rate in ESRD patients with digoxin [16]. However, giving neutral effects of digoxin on mortality rate in patients with CHF, it is inconclusive about the relationship between digoxin application and mortality rate in CAD patients with renal function decline. Digoxin has been recognized to improve clinical symptoms in RCTs and recommended as a supplement to treat symptomatic HF by ACC/AHA/ESC guidelines [3]. This analysis found that digoxin application had significant relationship with prolonged survive time regardless of patients with low GFR or not, but it had no significant relationship with mortality rate in Chinese older patients with CAD after adjustment in this analysis.

This analysis had several limitations. Firstly, patients had a median age of 86 years and relatively shorter survival time than younger patients in this analysis. Potential period over which drugs reduce mortality rate might not be reached in these patients. Thus, its results might not be simply generalized to younger patients. Secondly, this analysis enrolled patients with GFR < 60 ml/min/1.73m^2^, and they should not be assumed to be representative of general population with chronic kidney disease. Thirdly, the problem of residual confounding, which applies to all observational studies, could not be avoided by this analysis.

## Conclusion

The following conclusions were drawn in this analysis of patients with a median age of 86 years: 1) clopidogrel but not aspirin was beneficial in Chinese older CAD patients without low GFR rather than those with low GFR; 2) statins benefited for Chinese older CAD/CHF patients without low GFR rather than those with low GFR; 3) beta-blockers, CCBs, nitrates, ACEI/ARBs and digoxin had no significant relationship with mortality rate regardless of these patients with low GFR or not. These discoveries might offer some help for the therapy of Chinese older patients with cardiovascular/renal diseases.

## Data Availability

In attempt to preserve the privacy of patients, clinical data of patients will not be shared; data can be available from authors upon request.

## Abbreviations

ACEI/ARBs: Angiotensin-converting enzyme inhibitors/angiotensin receptor blockers
AF: Atrial fibrillation
BMI: Body mass index
CAD: Coronary artery disease
CCBs: Calcium channel blockers
CHF: Chronic heart failure
DBP: Diastolic blood pressure
FPG: Fasting plasma glucose
HDL-C: High-density lipoprotein cholesterol
LDL-C: Low-density lipoprotein cholesterol
MDRD: Modification of Diet in Renal Disease
SBP: Systolic blood pressure
